# Membrane-Targeting Antivirals

**DOI:** 10.3390/ijms26157276

**Published:** 2025-07-28

**Authors:** Maxim S. Krasilnikov, Vladislav S. Denisov, Vladimir A. Korshun, Alexey V. Ustinov, Vera A. Alferova

**Affiliations:** 1 Shemyakin-Ovchinnikov Institute of Bioorganic Chemistry, Miklukho-Maklaya 16/10, 117997 Moscow, Russia; makrlist@gmail.com (M.S.K.); vladdenya2004@gmail.com (V.S.D.); v-korshun@yandex.ru (V.A.K.); austinov@yandex.ru (A.V.U.); 2 Department of Chemistry, Lomonosov Moscow State University, Leninskie Gory 1-3, 119991 Moscow, Russia

**Keywords:** broad-spectrum antivirals, enveloped viruses, fusion inhibitors, photosensitizers, singlet oxygen, host-targeting antivirals, viral coating and egress

## Abstract

The vast majority of viruses causing human and animal diseases are enveloped—their virions contain an outer lipid bilayer originating from a host cell. Small molecule antivirals targeting the lipid bilayer cover the broadest spectrum of viruses. In this context, we consider the chemical nature and mechanisms of action of membrane-targeting antivirals. They can affect virions by (1) physically modulating membrane properties to inhibit fusion of the viral envelope with the cell membrane, (2) physically affecting envelope lipids and proteins leading to membrane damage, pore formation and lysis, (3) causing photochemical damage of unsaturated membrane lipids resulting in integrity loss and fusion arrest. Other membrane-active compounds can target host cell membranes involved in virion’s maturation, coating, and egress (endoplasmic reticulum, Golgi apparatus, and outer membrane) affecting these last stages of viral reproduction. Both virion- and host-targeting membrane-active molecules are promising concepts for broad-spectrum antivirals. A panel of approved antivirals would be a superior weapon to respond to and control emerging disease outbreaks caused by new viral strains and variants.

## 1. Introduction

For centuries, viral diseases have threatened the livelihoods and well-being of humankind. The 1918–1920 flu pandemic has killed tens of millions of people. Outbreaks of diseases caused by new viral strains, and especially the recent (March 2020–May 2023) COVID-19 pandemic, show that even with the 21st century healthcare conditions, viral infections can spread rapidly to hundreds of millions of people and cause infection, claiming millions of lives and threatening to set off a global civilizational crisis. The possibility of creating highly pathogenic artificial viral strains has now become real, which adds to fears for the future of humanity in the face of international tensions, interconfessional and ethnic conflicts. Therefore, despite ad-hoc political decisions, such as the March 2025 termination by the White House of COVID-related research grants [1], antiviral studies are still of great importance.

Vaccines represent one of the effective countermeasures against viral diseases, though they possess several inherent limitations. Vaccine development is a lengthy process: even with maximum speed and substantial funding, it takes many months to develop, properly demonstrate efficacy, and scale up production of an antiviral vaccine. Vaccines are therefore not applicable for controlling outbreaks caused by emerging strains. The high genetic variability of viruses reduces the effectiveness of vaccines developed for a particular strain: its mutation may render vaccines less effective [2]. In addition, for some viruses, the development of vaccines is difficult and still unsolved.

If new viruses/strains are identified, the development of an outbreak can be localized by sanitary and epidemiological measures. Nevertheless, effective antiviral drugs are required to reduce mortality among those who become infected. However, the complete development cycle for small-molecule drugs may take even longer than vaccine development. Therefore, the contemporary (formulated by Beckerman and Einav in 2015 [3] and subsequently developed by other researchers [4,5,6]) concept of promptly confronting new viruses is to accelerate *the repurposing of approved broad-spectrum antiviral drugs*. This remarkable concept has, however, one drawback—it has not yet been realized, as most antiviral drugs in clinical practice are not fully broad-spectrum drugs. During the COVID-19 pandemic, numerous attempts were made to repurpose pharmaceuticals (not only antiviral drugs) for the therapy of SARS-CoV-2 infection; the results of these studies are summarized in a number of experimental articles and reviews, e.g., in [7,8,9,10,11], with the overall success of the approach being assessed as rather modest [12]. Of particular interest is the repurposing of remdesivir and molnupiravir [13,14] along with the first FDA-approved COVID-19 therapeutic—the nirmatrelvir and ritonavir combination therapy [15] (its May 2023 approval coincided with SARS-CoV-2 infection transitioning to the post-pandemic phase). The primary antiviral action of nirmatrelvir, developed through structural optimization of a leading SARS-CoV-1 protease inhibitor [16], is enhanced by the co-drug ritonavir which inhibits nirmatrelvir metabolism. Although the general concept of drug repurposing is well-established for various diseases [17,18,19,20], the *broad-spectrum* activity of antivirals (vs. strain-specific activity) could simply increase the probability of successful repurposing.

Although the term *broad-spectrum antiviral* is rather ambiguous and variously interpreted, it generally means that a compound is capable of inhibiting several viruses and their strains. The similarity of viral targets underlies the broad-spectrum activity of a particular small molecule antiviral [21]. The development of broad-spectrum antiviral drugs has attracted global scientific interest. This approach would not only effectively combat known viruses, but could also be deployed against emerging strains, potentially preventing future pandemics. In this context, we focus on membrane-targeting antivirals capable of blocking various membrane-associated stages of viral replication, and thus being potentially applicable to all enveloped viruses.

## 2. Viruses and Viral Membranes

A virus (virion) is a nanoobject (20–200 nm in size), often spherical in shape. Virions contain genetic material inside and a capsid envelope or lipid membrane with integrated membrane proteins outside [22]. The taxonomy of viruses is based on their evolutionary relationships [23]. The classification of viruses proposed by David Baltimore—by type of genetic material (DNA or RNA, single- or double-stranded, (+)- or (−)-chain)—is useful for understanding replication [24]. Viruses that have an outer lipid membrane are called enveloped viruses. The latter include the vast majority of viruses dangerous for humans (influenza, SARS-CoV-2, and other respiratory viruses, herpes, many hemorrhagic fever viruses, rabies, smallpox, hepatitis C, HIV, etc.) and animals (African swine fever virus, feline infectious peritonitis virus, etc.).

**Figure 1:**
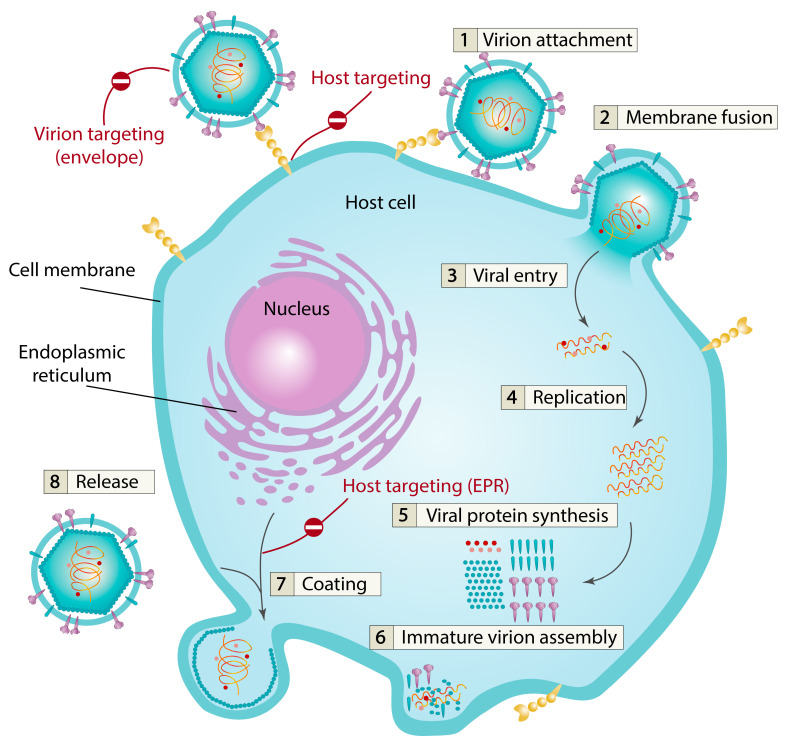
Schematic representation of the life cycle of enveloped viruses. Steps affected by membrane-active small molecules targeting virions and host cells are highlighted in red.

The lifecycle of viruses is carried out with the participation of the host cell. It begins with the recognition of cell receptors by the virus, followed by penetration of the virion’s contents into the cell, replication of viral proteins/enzymes and genetic material, assembly of new virions and their egress from the cell (Figure 1). Enveloped viruses acquire a lipid membrane from the host cell (from the endoplasmic reticulum, Golgi apparatus, nuclear or plasma membrane). Each antiviral drug affects its particular stage of the virus lifecycle (see examples in Table 1). For penetration of the genetic material of enveloped viruses into the cell, the necessary stages are virion attachment to the cell followed by fusion of the lipid membrane of the virion with the lipid membrane of the cell to form a pore [25,26,27]. Next stages are viral entry, replication, (reverse) transcription of viral genetic material along with translation of viral enzymes and capsomers followed by virion assembly, coating and egress.

The viral membrane typically contains multiple viral glycoproteins whose primary function involves host cell attachment and mediation of fusion between the viral envelope and the cell membrane [28]. Although fusion is mediated by receptor proteins, the lipid bilayer plays a crucial role. Its lipid composition can modulate the structure, organization, and dynamics of fusion proteins. Furthermore, during virion attachment to the host cell membrane, the lipid-binding domain of fusion proteins interacts with host regulatory lipids and controls membrane fusion [29]. The physical properties of viral membranes are largely determined by their components—various phospholipids, sphingolipids, cholesterol, diacylglycerol, etc. [29,30]. Additionally, an important component of viral membranes are lipid rafts—microdomains in the lipid bilayer containing high concentrations of sphingolipids, cholesterol, and various viral proteins [31]. These membrane regions are involved in virus−host cell interactions and may serve as targets for antiviral molecules [32,33]. Thus, both membrane lipid composition and protein lipidation (covalent lipid attachment to protein molecules) can influence viral entry processes.

Beyond enveloped viruses, quasi-enveloped viruses and extracellular vesicles represent additional viral exploitation strategies. Quasi-enveloped viruses are essentially non-enveloped virions cloaked in host-derived lipid membranes. In contrast to conventional enveloped viruses, these particles typically lack or contain minimal viral envelope proteins. For instance, hepatitis A and E viruses can exist as both naked and quasi-enveloped particles, enabling distinct cellular egress mechanisms [34]. These viruses acquire their membrane during virus-induced apoptosis, resulting in a structure identical to classical viral envelopes except for the absence of viral proteins. While membrane-targeting antiviral compounds (binding primarily to lipid bilayers or specific lipids) can disrupt these quasi-envelopes, their therapeutic efficacy remains limited. As noted, quasi-enveloped viruses typically coexist with naked virions unaffected by such drugs. Moreover, complete quasi-envelope destruction releases infectious naked virions capable of alternative entry pathways beyond membrane fusion.

Regarding extracellular vesicles, viruses co-opt these structures to regulate host RNA/protein expression profiles, modulating immune responses [35]. These membrane-bound vesicles, released by cells for intercellular communication, also derive their envelopes from host membranes. While membrane-targeting compounds can disrupt virus-hijacked vesicles, this approach offers limited therapeutic potential since vesicles primarily facilitate viral spread rather than serve as primary infection vehicles [35].

Table 2 shows examples of the main viruses, with an indication of the type of their genetic material.

Since envelope lipids are not encoded in the viral genome in any way (although the influence of viruses on cellular lipids has been discussed [36]), they are a promising target for broad-spectrum antiviral drugs (such drugs should be able to act on all enveloped viruses) [37,38,39,40,41,42]. The observed action of such broad-spectrum drugs allows for using simple-enough experiments to identify them as virus entry inhibitors or fusion inhibitors. It should be noted that fusion inhibitors can also include drugs that target receptor proteins. Thus, the membrane is a promising target for the development of antivirals of broadest possible coverage—against all enveloped viruses.

## 3. Research Methods

Studies of membrane-targeted antiviral activity mechanisms are frequently conducted using model membranes, and this method proves particularly suitable for investigating curvature effects. Various lipid bilayers or lipid vesicles can serve as model membranes—either freely suspended in solution or surface-immobilized, as well as in the form of nanoparticles: vesicles, liposomes, etc. [43]. The latter are most appropriate models for studying antiviral mechanisms. Their size resembles virions and, consequently, they exhibit similar curvature, enabling investigation of curvature-related effects.

To analyze changes in model membranes, the following methods have become widely adopted [43]:

− Fluorescence spectroscopy and microscopy—assess membrane permeability and morphological changes;
− Quartz crystal microbalance with dissipation monitoring—evaluate viscoelastic membrane properties;
− Electron spin resonance spectroscopy—monitor membrane organization;
− Electrochemical impedance spectroscopy—study membrane destabilization kinetics.

To study host cell-targeted mechanisms, classical biological analysis methods are used, including Western blotting, RT-qPCR, RNA sequencing, etc. These methods allow for the detection of specific viral and cellular proteins, as well as certain double-stranded nucleic acid sequences [44].

The combined use of these technologies with traditional experiments like “time of addition” allows for a comprehensive investigation of compound–lipid bilayer interactions. The “time-of-addition” experiment determines which stage of the viral life cycle is targeted by the test compound. This assay involves administering the investigational agent at different time points (pre-infection, during infection, and post-infection) to identify the specific phase of viral replication it inhibits.

Several compounds reviewed herein—particularly those modulating membrane physical properties—have only been tested on the aforementioned model membrane systems without demonstrated antiviral efficacy. Nevertheless, such studies provide critical insights into fusion mechanisms and reveal promising antiviral drug candidates.

## 4. Virion-Targeting Membrane-Active Antivirals

This section will provide a detailed description of agents targeting the viral membrane, ranging from disrupting its lipid bilayer rheology to pore formation and complete virion destruction. Additionally, it will briefly cover interactions with receptor proteins mediating fusion.

### 4.1. Membrane Properties Modulation

Viral inactivation and fusion inhibition can be achieved by modifying their physical/rheological properties—such as charge, curvature, fluidity, etc. Various peptides and small molecules can incorporate into the lipid bilayer, thereby disrupting it by physical means. In this case, membrane incorporation alters its physical properties through mechanical wedging, compaction, or reorganization of membrane components. Such effects often leave viral particle functionality unaffected until fusion initiation occurs.

**Figure 2:**
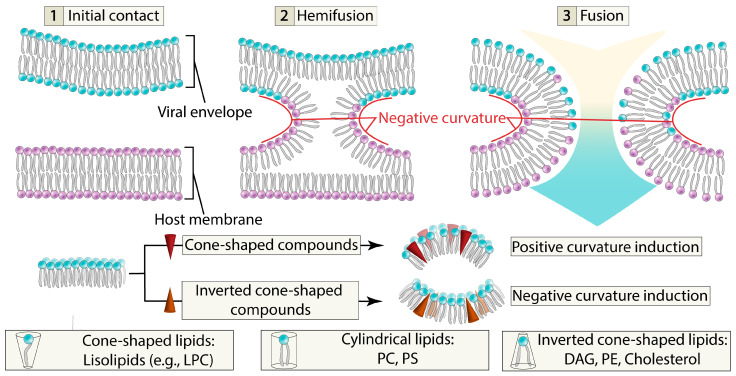
Schematic representation of negative curvature induction during the fusion process, influenced by cone-shaped and inverted cone-shaped compounds. LPC—lisophosphatidylcholine; PC—phosphatidylcholine; PS—phosphatidylserine; DAG—diacylglycerol; PE—phosphatidylethanolamine.

From the lipid bilayer point of view, viral membranes exhibit high positive curvature due to the small size of virions (~100 nm). However, during fusion of viral and host cell membranes, the viral envelope must overcome an activation barrier to transition from positive to negative curvature. Consequently, compounds promoting positive curvature inhibit fusion, while those promoting negative curvature facilitate it. This curvature modulation is primarily determined by molecular steric properties: cone-shaped molecules induce positive curvature, cylindrical ones maintain neutral curvature, and inverted cone-shaped molecules promote negative curvature [29] (Figure 2).

The physical properties of viral membranes are largely determined by their components—various phospholipids, sphingolipids, cholesterol, diacylglycerol, etc. Chemical modification or altered positioning of these lipids also inhibits fusion [29]. Beyond curvature, one can target the membrane charge or rigidity. Increased rigidity (reduced fluidity) impairs fusion by making conformational changes and restructuring more difficult. Regarding charge effects, negatively charged lipids like phosphatidylserine and phosphatidylglycerol inhibit membrane fusion due to repulsion from the negatively charged host cell membrane [45].

Examples of compounds affecting membrane curvature include various antimicrobial peptides (AMPs), such as plantaricin NC8 [46], kalata B1 [47], temporin L, and coronin-based peptides [48,49]. These peptides can inhibit a broad spectrum of enveloped viruses through direct non-specific interactions with viral surface components, demonstrating micromolar antiviral activity. Antimicrobial peptides (AMPs) constitute a key component of host immunity, exhibiting activity against pathogenic bacteria [50]. Regarding their mechanism of action, AMPs display considerable diversity, though they most commonly target pathogen membranes. Consequently, it is unsurprising that these peptides also demonstrate antiviral activity. AMPs can affect viruses not only by altering membrane physical properties, but also by disrupting its structural integrity, as will be further discussed in the relevant section. One proposed mechanism of action for temporin-based antimicrobial peptides appears to involve blocking early viral entry stages by interacting with surface carbohydrates, thereby disrupting the membrane and increasing its positive curvature. A recent study suggests that, in addition to altering membrane physical properties, temporin-based peptides may also bind to viral surface proteins or inhibit intracellular viral gene expression, producing a combined antiviral effect [48]. In turn, the lipopeptide derived from coronin-1, a mycobacterial envelope protein that inhibits phagosome-lysosome fusion, modulates membrane fusion by altering surface potential. Testing against influenza virus and murine coronavirus demonstrated both antiviral activity and fusion inhibition [49].

**Figure 3:**
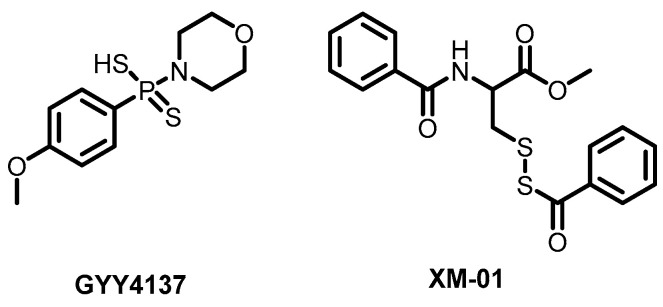
Structures of GYY4137 (a hydrogen sulfide donor) and one of the cysteine-based antiviral compounds XM-01.

Unexpected antiviral activity specifically against enveloped viruses was discovered in the past for hydrogen sulfide, or, more precisely, for precursor molecules that release H_2_S in the body, such as GYY4137 (Figure 3), sodium hydrosulfide, etc. [51]. These molecules demonstrate antiviral activity against SARS-CoV-2 and other enveloped viruses, leading authors to suggest potential interactions between H_2_S and viral membranes that disrupt their functions, though detailed mechanistic studies have not been conducted [52]. A breakthrough in this field came with the synthesis of a new series of a dozen of cysteine-based antiviral molecules (XM compounds). The most active compound, demonstrating antiviral activity (EC_50_) around 1 μM, XM-01 (Figure 3), affects the chemical composition of lipids by altering membrane properties deep within the hydrophobic region of the bilayer. It increases the phase transition temperature of the membrane, thereby modulating its fluidity. Apparently, sulfur plays a key role in membrane interactions. Antiviral activity was tested against a broad spectrum of enveloped viruses including Ebola virus, SARS-CoV-2, Nipah virus, herpes simplex virus-1, respiratory syncytial virus, human cytomegalovirus, vesicular stomatitis virus, and influenza A virus [53].

**Figure 4:**
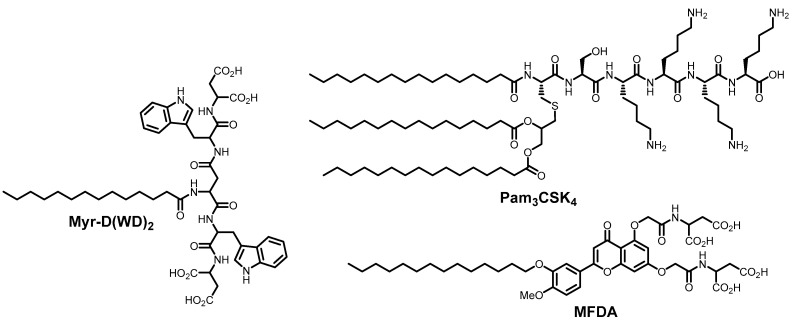
Amphipathic lipopeptide compounds possessing antiviral activity.

A large class of compounds that interact with membranes and alter their physical properties are cyclic and non-cyclic amphiphilic lipopeptides. These compounds contain a polar amino acid fragment linked to non-polar fatty acid tails, which appear to be responsible for interaction with the lipid bilayer [54,55]. Lipopeptide amphiphilic constructs containing aromatic residues between the fatty acid tail and ionogenic groups were recently synthesized and exhibit a slightly different mechanism of membrane interaction (Figure 4). Several flavonoids containing bulky substituents, e.g., myristyl flavonoid di-aspartic acid (MFDA), can intercalate into viral membranes, where these substituents wedge apart lipids, forming molecular nanodomains through hydrogen bonding between acidic substituents and π-π stacking between aromatic moieties. Such aggregates disrupt membrane interface properties, thereby impeding the transition to negative curvature and inhibiting fusion [56]. Despite demonstrating clear advantages for membrane wedging and property modulation, the reported compounds exhibit antiviral activity only at ~50 μM concentrations—a relatively modest efficacy threshold for small non-protein molecules. For comparison, various photosensitizers and other membrane-active small molecules demonstrate antiviral activity reaching submicromolar to subnanomolar ranges [57,58]. The lipopeptide Myr-D(WD)_2_ binds to the viral membrane and adopts a β-sheet-like structure [54]. The peptide self-organizes within the membrane, forming clusters and ordering the membrane interface. The branched architecture of such structures imparts positive curvature to the viral membrane, which negatively affects fusion between the viral and host cell membranes. The pronounced inhibitory effect of this lipopeptide has been utilized to protect cells against various viruses entering through both pH-dependent and pH-independent pathways: influenza A virus, mouse hepatitis virus, and human coronavirus [54].

**Figure 5:**
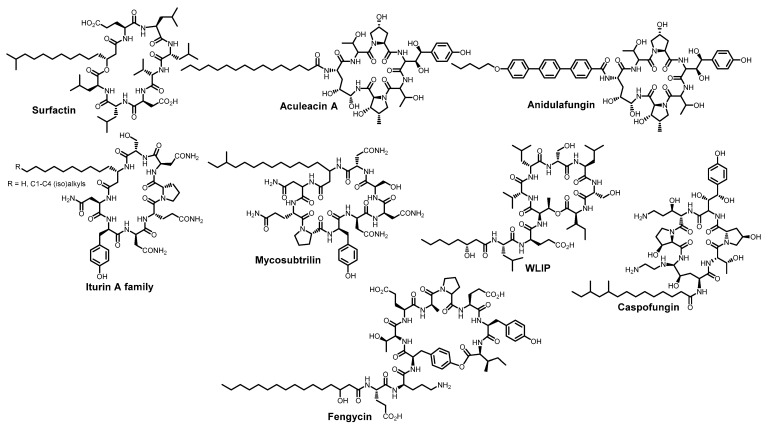
Cyclic lipopeptides possessing antiviral activity.

Another amphiphilic lipopeptide construct Pam_3_CSK_4_ (Figure 4) inhibits bunyavirus fusion, including Rift Valley fever virus, and demonstrates high antiviral activity with EC_50_ around 1 μM. The critical determinants of the Pam_3_CSK_4_ antiviral activity are its hydrocarbon residues, suggesting direct interaction with the viral membrane [59]. Natural and synthetic amphiphilic cyclopeptides (Figure 5) also exhibit antiviral activity against enveloped viruses. The antiviral activity of surfactin is well-documented [60,61,62]. Its mechanism of action involves interaction with the viral membrane, hindering the formation of negative curvature and thereby inhibiting fusion. There is also evidence that surfactin can disrupt the integrity of the SARS-CoV-2 viral envelope. Apparently, the mechanism of action of this cyclopeptide is concentration-dependent: at low doses, surfactin incorporates into the viral membrane and alters its physical properties, while high concentrations lead to disruption of the lipid bilayer [61,62]. Later, Shekunov et al. demonstrated that other lipopeptides, aculeacin A, andidulafungin, inurin A, and mycosubtrilin (Figure 5) showed pronounced activity against a SARS-CoV-2 strain in Vero cells with selectivity indices of 47, 8, 12, and 41, respectively [55]. Then, Hoste et al. added WLIP, fengycin, and caspofungin to the panel of anti-SARS-CoV-2 lipopeptides [63]. Similarly to surfactin, all these compounds are capable of interacting with the viral envelope, altering lipid packing, membrane tension and its curvature, but usually the mechanism of action is described rather unconvincingly. A recent review also examined the membrane-targeted (including antiviral) effects of various other *Bacillus* lipopeptides [64]. However, it should also be noted that in the aforementioned study by Shekunov et al., caspofungin demonstrated neither cytotoxicity nor activity, while surfactin exhibited significant cytotoxicity.

It was discovered that the amphipathic α-helical (AH) antiviral peptide derived from the N-terminus of hepatitis C virus NS5A protein can bind to membranes with high positive curvature. The AH peptide associates with nanoscale vesicles that, like virions, possess highly curved and stretched membranes due to their extreme curvature. These findings suggest this peptide’s affinity for viral membranes, leading to their stretching [65].

Certain natural alkaloids can also affect membrane packing, particularly by altering lipid phase transition temperatures. Membrane lipids can exist in different phases or, metaphorically speaking, states of aggregation. This variability stems from the distinct environmental requirements of membrane proteins—some require more fluid environments while others function optimally in denser packing. Consequently, membrane microdomains may exhibit phase heterogeneity and even undergo phase transitions at specific temperatures, depending on their local composition [66]. These modifications, demonstrated in model membrane studies, would be expected to significantly inhibit fusion—as has been shown for MERS-CoV, SARS-CoV, and SARS-CoV-2 viruses [67].

**Figure 6:**
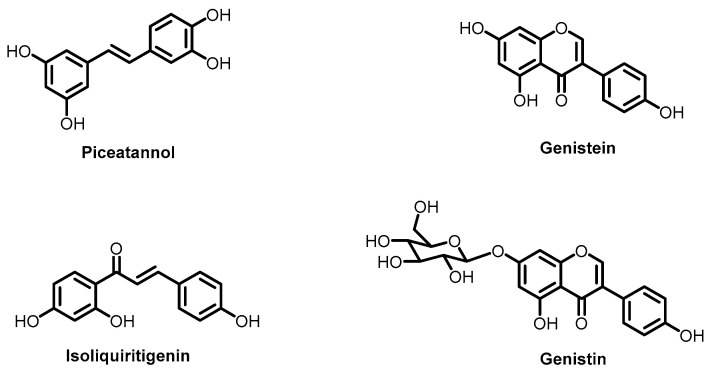
Polyphenols affecting membrane curvature.

The next class of low molecular weight compounds includes various polyphenols, e.g., piceatannol, genistein, isoliquiritigenin, genistin, etc. (Figure 6) [68,69,70,71]. Polyphenols are frequently tested as mixtures since they are isolated from natural extracts: *Artemisia* species [71], *Cassia abbreviata* [68], etc. The mechanism of action for individual components in these mixtures is often not fully characterized and remains hypothetical. The intercalation of polyphenols between lipid headgroups induces disordering and creates positive bending stress. This should suppress membrane fusion by increasing the energy required to form high-negative-curvature fusion intermediates. More hydrophobic molecules containing fewer hydroxyl groups can penetrate deeper into the membrane and cause greater lipid layer disordering [69]. This hypothesis is supported by the demonstrated antiviral activity of various plant polyphenols against Ebola and Marburg viruses [70]. Due to their amphiphilic properties, these compounds efficiently intercalate into lipid membranes and, as mentioned, alter the bilayer’s biophysical properties.

Another fundamental study investigates the mechanism of interferon action—a class of natural signaling proteins that activates the body’s immune response against various pathogens, including viruses [72]. Elucidating interferon mechanisms of action may reveal novel pathways for antiviral activity implementation. One of the interferon-stimulated genes, cholesterol 25-hydroxylase (CH25H), is induced by SARS-CoV-2 infection both in vitro and in COVID-19 patients. CH25H converts cholesterol to 25-hydroxycholesterol (25HC), which exhibits broad anti-coronavirus activity by blocking membrane fusion [73]. Furthermore, 25HC inhibits SARS-CoV-2 infection in lung epithelial cells and viral entry into human lung organoids. Mechanistically, 25HC inhibits viral membrane fusion by activating endoplasmic reticulum-localized acyl-CoA: cholesterol acyltransferase (ACAT), leading to depletion of available cholesterol in the plasma membrane. Cholesterol deficiency not only affects membrane curvature and fluidity, but also alters the positioning of fusion-mediating spike proteins in the cellular membrane. Thus, 25-hydroxycholesterol itself represents a promising antiviral agent [73].

The diversity of both small molecules and peptide-based compounds that modulate physical properties of viral membranes stems from their high efficacy as antiviral agents and the availability of methods to study their mechanisms of action. High selectivity is achieved through significant differences between viral and cellular membrane curvature, as well as the size disparity between virions and host cells. Furthermore, the transition from positive to negative curvature represents a critical process in the viral life cycle, essential for successful fusion with host cells—in contrast to host membranes, which do not require negative curvature formation.

### 4.2. Membrane-Lytic Compounds

Another extensive membrane-targeting mechanism of antiviral activity involves complete destruction of the viral envelope. Various antiviral peptides or peptidomimetics, which act on different targets within the viral membrane, often serve as examples of such compounds. The detailed mechanism remains under active investigation and appears to be based on interactions with the lipid bilayer of the virion envelope. Most commonly, researchers focus on active sites within the lipid bilayer where antiviral peptides bind. Consequently, the mechanism of peptide-membrane binding, in contrast to the mechanism of membrane disruption itself, has been well-characterized.

An example of antiviral peptides are labyrinthopeptides (LPs) [32,33,74], which exhibit submicromolar antiviral activity by disrupting viral envelopes and inducing virolysis. The prerequisite for labyrinthopeptide-induced lysis is the presence of phosphatidylethanolamine (PE) in the membrane composition, which specifically binds to LPs. Generally, PE constitutes approximately 15–25% of total phospholipids in mammalian cells. Labyrinthopeptides are particularly active against lipid rafts—microdomains in the lipid bilayer that contain high concentrations of non-lipid components: proteins, cholesterol, sphingolipids, etc. [32]. These rafts often possess elevated concentrations of phosphatidylethanolamine, making them primary targets for labyrinthopeptides.

The low cytotoxicity of LPs is explained by the asymmetric distribution of phosphatidylethanolamine in cellular membranes. PE lipids are located in the inner endoplasmic membrane layer but are absent from the outer exoplasmic layer. Consequently, labyrinthopeptides cannot bind to host cells. In contrast, viruses acquire their envelopes from intracellular compartments. For instance, flaviviruses bud from the endoplasmic reticulum, which lacks this membrane asymmetry. In the ER, PE lipids are distributed on both inner and outer membrane leaflets, and this distribution is consequently inherited by flaviviruses [32].

Another example is the cationic peptide derived from the cowpox virus protein CPXV012 [75]. This peptide has been reported to specifically bind to and disrupt membranes containing the anionic phospholipid phosphatidylserine, an important component of many viral membranes. The peptide demonstrates activity against poxviruses, herpes simplex virus, hepatitis B virus, HIV, and Rift Valley fever virus. However, infections caused by non-enveloped viruses, such as Coxsackievirus B3 and adenovirus, remain unaffected by CPXV012.

Recent studies have also reported antiviral activity data for various other proteins and peptides, including antimicrobial peptides [76,77], melittin [78] and various peptidomimetics [79]—non-protein compounds containing protein-like structural elements. While all these agents demonstrate activity against multiple enveloped viruses by targeting and disrupting viral membranes, their exact mechanisms of interaction require further elucidation. Antimicrobial peptides exhibit broad-spectrum antiviral effects, likely through initial binding to membrane proteins followed by direct disruption of the lipid bilayer. Notably, certain proteins like melittin display both potent antiviral activity and considerable cytotoxicity, reflecting their natural origin as biological toxins.

**Figure 7:**
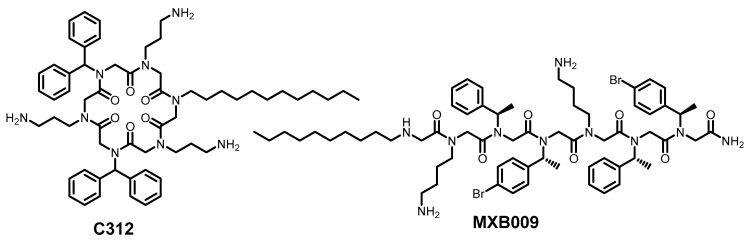
Membrane-lytic amphiphilic peptidomimetic/peptoid oligomers.

The amphiphilic peptidomimetic oligomers MXB009 and C312 (Figure 7) likewise exhibited antiviral activity against Zika, Rift Valley fever, and chikungunya viruses [80]. Liposome leakage assays revealed that the antiviral mechanism depends on membrane intercalation and component disruption. Phosphatidylserine-containing membranes showed significantly greater susceptibility to MXB009, while pure phosphatidylcholine liposomes remained unaffected. Consistently, chikungunya virus, whose membrane is phosphatidylserine-enriched, demonstrated heightened MXB009-mediated inactivation.

To conclude the discussion on antiviral peptides targeting viral membranes, we should mention a recent review examining arginine’s antiviral properties [81]. Research in this field identifies this amino acid as a key binding motif in many antiviral peptides. Studies confirm that arginine likely inhibits viral fusion, though the exact mechanism remains unclear. The authors propose several hypotheses: arginine may interact with receptor proteins, bind directly to viral membrane lipids, or form pores in the viral envelope—all mechanisms that would compromise membrane integrity. Since the antiviral effect is observed exclusively against enveloped viruses, the authors favor direct membrane interaction as the primary mode of action [81].

Antiviral peptides selectively bind to specific domains and structural motifs of viral membranes, which inherently limits their spectrum of activity—they remain ineffective against viruses lacking these target features. While these peptides demonstrate high submicromolar antiviral potency, a critical consideration emerges: compared to small-molecule agents (e.g., photosensitizers), peptides have significantly higher molecular weights. Consequently, mass-for-mass, larger quantities are required for equivalent efficacy. Furthermore, peptide synthesis is complex and costly, and large-scale isolation from natural sources often proves economically impractical.

**Figure 8:**
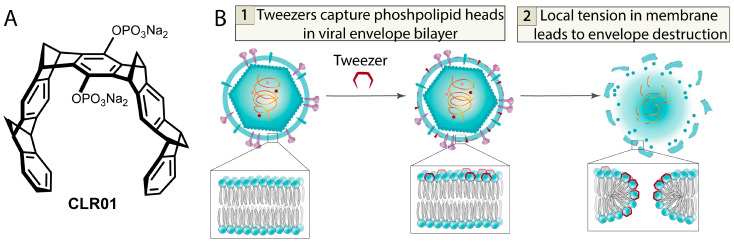
(**A**) Structure of molecular tweezer CLR01. (**B**) Schematic representation of viral envelope lysis mediated by the molecular tweezer effect.

A completely distinct and unique class of compounds that disrupt viral membranes are so-called molecular tweezers [57,82,83,84]. These are small molecules bent in a specific way, resembling forceps or tweezers (Figure 8A). In addition to the “tweezer” part, such molecules contain a hydrophilic moiety on a short linker. The primary mechanism of action of tweezers is based on their unusual shape. The tweezers first capture the lipid head, which causes a horizontal orientation of lipids within their enclosed cavities. This process promotes the insertion of the tweezer into the polar region of the outer membrane layer and increases local tension in the latter. Such restructuring ultimately ruptures viral membranes into fragments and inactivates viral particles (Figure 8B). Tweezers exhibit low cytotoxicity because the curvature of cells is significantly lower than that of viruses, making it difficult to capture the lipid head of host cells. Additional mechanistic studies demonstrate that the tweezer-like shape is indeed critical for antiviral activity. If an additional substituent blocking the tweezer cavity is introduced into the molecule, it loses its ability to bind membranes [82]. Tweezers show low-micromolar and submicromolar (EC_50_ ~ 1 μM) activity against a broad spectrum of enveloped viruses, including HIV, SARS-CoV-2, Zika virus, Ebola virus, herpes simplex viruses 1 and 2, and influenza A virus. Inhibitory activity against SARS-CoV-2 has also been confirmed in vivo [57].

Thus, tweezers selectively target viral membranes directly, disassembling them into individual lipids and completely destroying the viral envelope. Their cytotoxicity is significantly lower than their antiviral activity, which is attributed to the difference in curvature between the virion envelope and cell membranes. These molecules represent promising antiviral agents and have already demonstrated activity against a broad spectrum of viruses. As a drawback, one might mention only their low solubility in aqueous media due to the presence of the non-polar “tweezer” moiety.

To conclude our discussion of methods for complete viral membrane disruption, we must mention surfactants. A recent review focused on the effects of surfactants on enveloped viruses [85]. Due to the structure of viral membranes—like any other lipid bilayer-based membrane—they are susceptible to surfactants. Depending on the surfactant composition, it is possible to achieve either complete membrane dissolution with release of viral genetic material or partial bilayer damage through extraction of specific lipids or proteins. Moreover, surfactants are already used in antiviral applications, though primarily in disinfectants and personal hygiene products—this method is suitable for complete destruction of enveloped viruses on various surfaces, whether objects or human skin. Along with viral membranes, surfactants can also effectively dissolve other cell membranes, making their use for targeted therapy within the body challenging. Thus, although surfactants act on all lipid membranes without selectivity, their simple production and ability to completely destroy viral envelopes make them valuable for surface disinfection.

### 4.3. Photosensitizers

**Figure 9:**
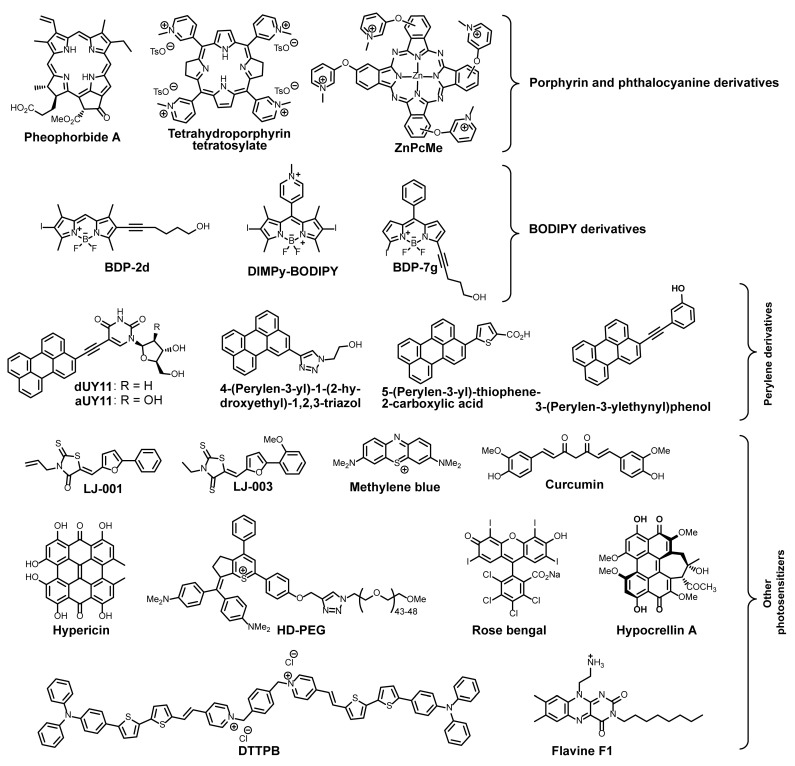
Structures of known antiviral photosensitizers [58,97,98,99,100,101,102,103,104,105,106,107,109,114,116,118,123,124,125,126,127,128,129,130,131,132,136,137,138,139,141,143].

To inhibit the fusion of viral and host cell membranes, complete destruction of the viral envelope is not always necessary—even minor disruptions to the membrane structure can suffice. A promising class of compounds exhibiting antiviral activity through viral membrane disruption and fusion inhibition are photosensitizers (PSs). Photosensitizers are molecules capable of generating reactive oxygen species (ROS) upon light exposure. The antiviral action of photosensitizers has been well studied [86,87,88,89,90,91,92,93,94,95,96]. As antiviral agents, aromatic organic compounds modified with polar groups to enhance aqueous solubility are most commonly used. Key examples of photosensitizers demonstrating antiviral activity include porphyrins [97,98,99,100,101,102,103,104,105,106,107,108], phthalocyanines [109,110,111,112,113], curcumins [114], flavins [115,116,117,118], hypocrellins [119,120] and hypericins [121,122,123], perylene derivatives [58,124,125,126,127,128,129,130,131], BODIPY dyes [132,133,134], and other aromatic conjugated compounds [135,136,137,138,139,140,141,142] (Figure 9).

**Figure 10:**
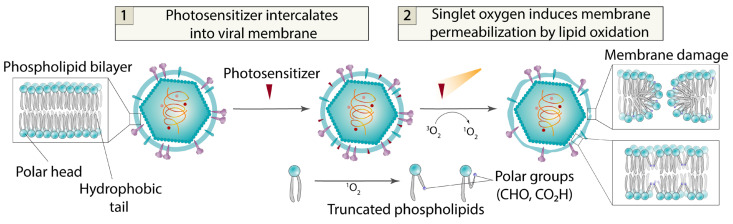
Mode of action for membrane-active photosensitizers (singlet oxygen photogenerators).

The mechanism of the antiviral activity of photosensitizers (PSs) is still under investigation. However, it is known that the dye molecule incorporates into the viral membrane via its non-polar aromatic core, which generates singlet oxygen. This singlet oxygen, in turn, oxidizes unsaturated double bonds in fatty acids [144,145,146]. The polar groups formed during oxidation migrate to the membrane surface, thereby disrupting its structure [147,148]. Due to the small size of viral particles (~100 nm) and, consequently, the high curvature of their envelope, even minor disruptions to the viral membrane significantly inhibit its fusion with host cells (Figure 10). Furthermore, photosensitizers often exhibit remarkably high antiviral activity, reaching subnanomolar efficacy levels (from 3 nM to 1 μM for perylene derivatives) [58,149]. The hypothesis about such a mechanism of antiviral action of membrane-active photogenerators of singlet oxygen was explicitly expressed more than 10 years ago [136,150,151]. Microscopy confirms the detrimental effect of singlet oxygen on the virion membrane [152,153].

Recent studies have revealed that a crucial factor in the photosensitization mechanism of antiviral activity is the presence of a hydrophilic head group attached via a long linker. When the photosensitizer’s non-polar core intercalates into the membrane, the polar head remains at the surface, interacting with charged phosphate esters, thereby anchoring the PS more firmly within the lipid bilayer. Furthermore, linker length appears to play a significant role—the linker must be of appropriate size to allow the aromatic core to freely migrate to the membrane surface. This migration event likely facilitates interaction with dissolved oxygen in the extracellular space, leading to singlet oxygen generation [132]. BODIPY-based photosensitizers incorporating the described structural motifs not only demonstrate high in vitro antiviral activity, but have also been validated in vivo. A mouse cohort infected with tick-borne encephalitis virus pre-treated with BDP-2d dye (Figure 9) and exposed to green light showed no infection signs throughout a one-month observation period. In stark contrast, the control group infected with a virus that is not treated by a photosensitizer experienced 100% mortality by day 8 [132].

The primary limitation of photosensitizing agents is their requirement for illumination to generate singlet oxygen. In practical applications, photosensitizers (PSs) can only be utilized for treating superficial infections. The activity of PSs without irradiation is typically 2–3 orders of magnitude lower than their light-induced activity and often comparable to their cytotoxicity. The development of antiviral photosensitizers absorbing in the near-infrared range enables greater penetration depth of irradiation due to tissue transparency to infrared light. Biological tissues typically transmit near-infrared light to average depths of 1–2 mm, with maximum penetration reaching 5 mm [154]. While this penetration depth remains insufficient for effective systemic photosensitizer activation, it significantly enhances the treatment area and therapeutic efficacy of topical photosensitizing agents.

Certainly, photosensitizers may also interact with healthy cell membranes; however, their cytotoxicity is generally 2–4 orders of magnitude lower than their antiviral activity. This is primarily attributed to the small size of viruses and the high curvature of their membranes—even minor damage to the virion envelope leads to significant structural alterations that adversely affect the fusion process. Furthermore, in contrast to viruses, host cells possess various membrane repair mechanisms capable of correcting minor damage.

It is particularly noteworthy that some photosensitizers can affect not only the lipid bilayer of viral envelopes, but also various viral proteins. For instance, the HIV-1 envelope protein may serve as a potential target for porphyrins. One possible mechanism of viral entry inhibition by porphyrins may involve their interaction with viral proteins. HIV-1 cellular entry is mediated by specific interactions between viral glycoprotein 120 (gp120) and CD4 receptors. This interaction induces conformational changes that facilitate virion binding to chemokine coreceptors. It also triggers conformational alterations in the viral transmembrane protein, initiating fusion with the cellular membrane and enabling viral entry and genome delivery. This entry cascade is inhibited through light-dependent binding of porphyrin derivatives to viral proteins, although the detailed mechanism remains uncharacterized [155].

In some cases, experimental evidence and mechanistic studies confirm that membrane deformation alters its rigidity and curvature, consequently inhibiting fusion. For example, the activity of pheophorbide A has been demonstrated against SARS-CoV-2 and MERS-CoV viruses. Pheophorbide A operates exclusively through a light-dependent photosensitization mechanism, incorporating into the lipid bilayer and damaging it. These changes reinforce the viral membrane, increasing its stiffness and inhibiting fusion [102]. Another notable example includes curcumin and its derivatives [114,156]. These photosensitizers have long shown antiviral activity against a broad spectrum of viruses, though their numerous potential molecular targets complicate mechanistic studies. Curcumin derivatives can affect both the host cell (suppressing replication through various pathways) and the virion itself (by binding to the membrane or viral proteins). Studies with hepatitis C and Zika viruses demonstrated that membrane-bound curcumin alters membrane fluidity, negatively impacting fusion [114]. Although the literature does not explicitly mention this, we propose that membrane damage is light-mediated and based on a photosensitization mechanism.

It has long been established that hypericin’s biological activity is associated with singlet oxygen photoproduction in lipid membranes. Consequently, studies have examined both its behavior in bilayers and singlet oxygen generation therein [157]. Quantum chemical calculations confirmed that hypericin molecules indeed intercalate into lipid bilayers, positioning adjacent to double bonds of unsaturated fatty acids—primary targets of singlet oxygen [158]. However, hypericin demonstrates antiviral activity even in darkness (albeit significantly reduced), suggesting an alternative mechanism distinct from photosensitization [122]. Current hypotheses propose that through its affinity for viral membranes, hypericin may mechanically alter membrane properties, explaining its dark activity—though research in this area remains ongoing [159].

Riboflavins are established singlet oxygen photogenerators. A recent study synthesized amphiphilic alkyl riboflavin derivatives and demonstrated their antiviral activity against mouse hepatitis enveloped virus. Notably, the authors contend that flavins exhibit activity against both enveloped and non-enveloped viruses, with their mechanism involving singlet oxygen-mediated oxidation of viral nucleic acids [118]. Separate research documents riboflavins’ capacity to inhibit SARS-CoV-2 viral proteases [160]. However, riboflavin-induced photochemical disruption of cellular lipid bilayers has also been demonstrated [117], preserving the plausibility of membrane-associated antiviral mechanisms. Current evidence suggests that these derivatives may act multimodally, employing one or several concurrent mechanisms.

Highly promising antiviral data have been demonstrated by a class of photosensitizers based on aggregation-induced emission (AIE). The membrane-active photosensitizer DTTPB (Figure 9) features a hydrophilic head and two hydrophobic tails mimicking the phospholipid structure in biological membranes. It exhibits broad absorption covering the entire visible light spectrum with high molar absorptivity, coupled with efficient reactive oxygen species generation—making it an excellent candidate for PDT. Similar to other photosensitizers described in this review, DTTPB and its analogues interact with viral membranes to disrupt their integrity. Its potent antiviral activity against enveloped viruses, including SARS-CoV-2 and rabies virus, has been experimentally confirmed [141,142].

Notably, singlet oxygen appears most effective for damaging enveloped virus membranes. Lipid peroxidation of virion envelopes mediated by free radical generators produced significantly weaker antiviral effects, while addition of radical-scavenging antioxidants showed negligible impact on antiviral activity [161].

A summary of the photosensitization mechanism of antiviral activity reveals the following key advantages:

− Exceptionally high antiviral activity, reaching subnanomolar efficacy levels;
− Low cytotoxicity due to the presence of lipid reparation system in the cell;
− Broad-spectrum potential—photosensitizers may theoretically target all enveloped viruses.
− Primary limitations include the following:
− Light-dependent action with no dark activity mechanism;
− Poor aqueous solubility resulting from the essential non-polar aromatic core required for both singlet oxygen photogeneration and membrane intercalation.

## 5. Host Cell-Targeted Antiviral Drugs

The mechanism of action of antiviral compounds targeting host cells is based on two key advantages:

1. These agents do not directly interact with viral proteins, thereby reducing the likelihood of drug-resistance mutations emerging.
2. They can target host receptor proteins common to multiple viruses, enabling broad-spectrum activity.

This section will examine compounds whose antiviral effects are mediated through host–cell interactions, yet remain directly or indirectly linked to viral membrane processes.

### 5.1. Host Cell Receptor Proteins

Targeting host cell receptor proteins responsible for viral fusion is presumed to be more effective because these proteins are obviously not encoded in the viral genome, making resistance development more challenging. While numerous compounds interact with cellular receptor proteins, most of them are not directly membrane-associated and thus fall outside the scope of this review [26,39,162,163,164,165,166,167]. The primary research focus in this field centers on isolating and modifying key structural domains of viral spike proteins to inhibit viral-cellular receptor protein–protein interactions. These viral proteins mediate host cell attachment and membrane fusion. Thus, peptides containing core receptor fragments bind to the corresponding cell surface proteins and block them, leaving no space for actual virion proteins to interact.

**Figure 11:**
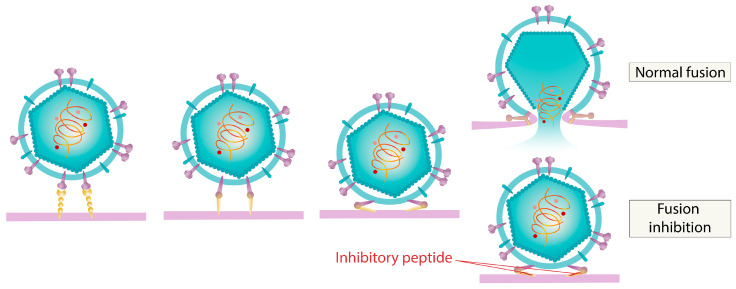
Inhibitory peptide-mediated fusion inhibition.

Examples of such compounds include multiple peptides derived from the HR2 domain of coronavirus fusion spike proteins, which exhibit potent antiviral activity by blocking receptors on healthy cell surfaces [168,169,170]. Optimization through amino acid sequence variation of the selected fragments, along with modifications via lipid or sugar moiety incorporation, has enabled the achievement of subnanomolar antiviral potency [170]. Another example is the compound AEEA-16, a peptidomimetic that replicates key functional groups of the MERS-CoV receptor protein, which also effectively inhibits fusion through a similar mechanism and shows activity with an EC_50_ value of 5 μM [171]. The mechanism of action of such peptides is shown in Figure 11.

A pivotal 2024 coronavirus study employed stem cell-derived airway organoids that closely mimic human respiratory tracts. Researchers infected these organoids with SARS-CoV-2 to investigate how lipid-modifying statins affect viral pathogenesis, gene expression, and intracellular trafficking of the SARS-CoV-2 entry receptor. Statins manipulate lipid homeostasis by reducing cholesterol levels. This membrane modification triggered relocalization of the viral entry receptor angiotensin-converting enzyme 2 (ACE2)—responsible for SARS-CoV-2 virion fusion—from the plasma membrane to the cytoplasm, consequently reducing viral entry [172].

Finally, cholesterol modification can be used to target membrane-disrupting peptides that block the helix bundle heptad repeat membrane fusion mechanism [173]. The SARS-CoV-2 surface glycoprotein binds to the host cell receptor protein, leading to the formation of a six-helix bundle (6-HB) that pulls together and subsequently fuses the host and viral membranes. A cholesterol-modified cross-linked lipopeptide decoy incorporates into the cell membrane and disrupts 6-HB assembly by sequestering the viral protein, thereby blocking fusion of viral and host cell envelopes. The cholesterol moiety serves as an anchor, intercalating into the lipid bilayer and securing the lipopeptide within the membrane.

Antiviral agents that interact with host cell receptor proteins demonstrate high antiviral activity and represent promising antiviral therapeutics. Current research on compounds with this mechanism of action generates significant interest, though most studies focus on protein–protein interactions that are not directly related to either host cell membranes or viral membranes.

### 5.2. Inhibition of Virus Coating

Drugs targeting viral replication inhibition demonstrate high efficacy by acting on DNA and RNA polymerases, transcription and translation components, and post-translational processes. As mentioned previously, enveloped viruses acquire their membrane from the host cell by budding through it after translation during release into the extracellular space.

**Figure 12:**
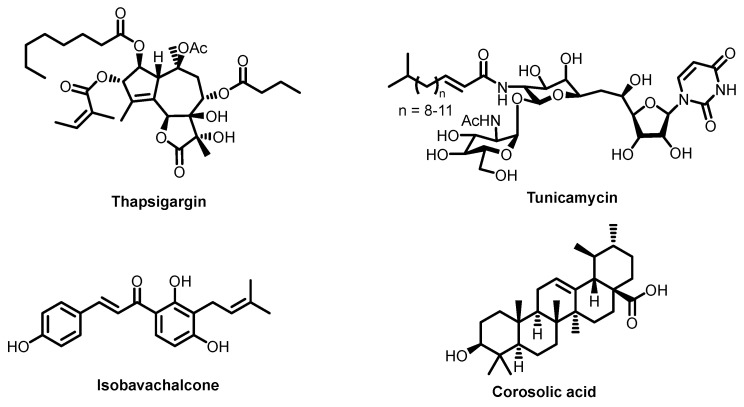
Structures of thapsigargin, tunicamycin, isobavahalcone, and corosolic acid.

The recently discovered molecule thapsigargin (Figure 12), isolated from dill, has demonstrated high antiviral activity against enveloped viruses. Although its complete mechanism remains under investigation, thapsigargin is known to affect viral assembly in host cells by inhibiting envelope formation and viral release [44,174,175,176,177,178,179,180,181,182]. Thapsigargin’s primary target is the endoplasmic reticulum (ER), responsible for protein folding and lipid production for various membranes. It acts on the ER calcium ATPase, inducing ER stress. Essentially, this activates the cell’s immune response—the stressed ER becomes activated and combats the virus through multiple pathways. ER stress-mediated metabolic changes affect numerous processes including translation and post-translational modifications. This occurs through alterations in protein folding patterns and lipid synthesis. The ER membrane becomes reinforced through compositional changes, while lipid droplets—which drift in the cytoplasm and are synthesized by the ER—also undergo compositional modifications [180]. Both the ER membrane and the lipid droplets can be utilized by viruses for envelope assembly; changes in their composition inhibit viral “wrapping” in membranes. Additionally, disrupted protein folding impacts antiviral activity. For instance, ubiquitination of the viral M2 membrane protein plays a crucial role in producing infectious virions by coordinating efficient viral genome packaging during virus-induced cell death [182]. ER stress suppresses lysine residue ubiquitination in viral proteins, thereby inhibiting viral production. Notably, ER stress activation is generally not dangerous for healthy cells—various stress response and control mechanisms exist that cells successfully employ. This is evidenced by thapsigargin’s low cytotoxicity, indicating that healthy cells can manage ER stress autonomously. While thapsigargin’s mechanism is not fully understood, current evidence suggests that it may inhibit fusion, though its primary effect involves disrupting viral replication. By triggering ER stress, thapsigargin simultaneously impacts multiple cellular processes—including viral membrane formation through effects on both viral proteins and membrane composition.

Thapsigargin has demonstrated broad antiviral efficacy against multiple enveloped viruses including porcine coronaviruses, transmissible gastroenteritis virus (EC_50_ 58 nM), [44], human coronaviruses (SARS-CoV-2, MERS-CoV, EC_50_ 7 μM) [174,178,179], and respiratory viruses (influenza A virus, respiratory syncytial virus, EC_50_ 65 nM) [181,182]. In this study, the authors propose that its mechanism suggests potential activity against all enveloped viruses.

Similar to thapsigargin, other compounds like tunicamycin (Figure 12) [175] also exhibit endoplasmic reticulum (ER) stress activation, though their mechanisms are less understood and differ substantially from thapsigargin. Other molecules affecting viral membrane assembly in host cells include isobavachalcone and corosolic acid (Figure 12), which show activity against Japanese encephalitis virus (a flavivirus) [183]. Flavivirus infection hijacks host lipid metabolism to remodel viral envelopes, creating a specialized lipid environment favorable for viral replication, assembly, and release. The non-structural Japanese encephalitis virus proteins NS1 and NS2A suppress 5′-adenosine monophosphate (AMP)-activated protein kinase (AMPK) activation. This reduces AMPK expression and triggers lipid synthesis upregulation. Isobavachalcone and corosolic acid stimulate AMPK by binding to its allosteric active site, thereby inhibiting virus-essential lipid synthesis and ultimately reducing viral load. Thus, these compounds affect viral membrane assembly, but, in contrast to thapsigargin, solely through lipid production inhibition.

This review has already discussed photosensitizers’ effects on both viral membrane physical properties and virion receptors. Recent studies additionally demonstrate that porphyrin derivatives containing long alkyl tails can suppress viral activity post-fusion by targeting replication. These porphyrin compounds accumulate in intracellular membranes, including lysosomes and the Golgi apparatus. Although the detailed mechanism remains uncharacterized, light-activated photosensitizers are proposed to damage host cell internal membranes that viruses exploit for envelope formation, thereby inhibiting viral particle assembly and release [100]. Such porphyrins exhibit subnanomolar antiviral activity against herpes simplex virus. Notably, at higher concentrations, membrane damage-mediated fusion inhibition also contributes to their antiviral effects.

Antiviral molecules inducing ER stress, such as thapsigargin and tunicamycin, combine high antiviral activity with minimal cytotoxicity. This occurs because their mechanism fundamentally activates and enhances the cell’s intrinsic immune response rather than directly modifying or destroying viral or cellular compartments. Such multimodal action simultaneously targets multiple viral replication processes, with viral budding being just one affected stage. This broad activity profile substantially reduces viral resistance development potential, making these compounds promising broad-spectrum therapeutic candidates.

**Figure 13:**
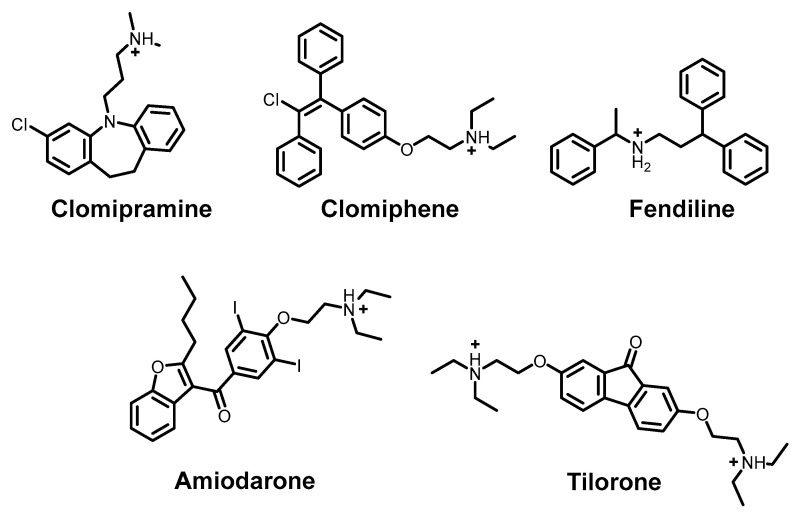
Example structures of cationic amphiphilic potential antiviral drugs (CADs) [186,187,188,189,190].

Cationic amphiphilic drugs (CADs) (Figure 13) demonstrating high in vitro antiviral activity against SARS-CoV-2 warrant special consideration. While some progressed to clinical trials, their mechanism of action remains ambiguous. Current evidence suggests that their antiviral effects stem from phospholipidosis—the formation of vesicle-like structures through disruption of cellular lipid metabolic pathways. Though phospholipidosis mechanisms remain incompletely understood, these CADs accumulate in host cell compartments (lysosomes/endosomes), disrupting lipid metabolism. As cationic amphiphiles, they become trapped in lysosomes, interacting with negatively charged intralysosomal vesicles—primary platforms for sphingolipid degradation [184]. While this process impacts viral life cycles by interfering with replication, membrane assembly, and egress, it represents a therapeutic dead-end. Phospholipidosis-inducing compounds may exhibit potent apparent antiviral activity, but their inherent cytotoxicity precludes clinical application [185]. Thus, while CADs effectively target host cells and demonstrate in vitro antiviral effects, they constitute an untenable branch of antiviral drug development.

## 6. Conclusions

Targeting membrane-associated stages of enveloped virus replication with small molecules holds promise for the development of broad-spectrum antiviral therapeutics. However, the complexity and multicomponent nature of membranes, combined with the diverse and multifactorial antiviral mechanisms exhibited by these molecules, complicate the detailed elucidation of their modes of action. Nevertheless, drugs developed through this strategy may have significant potential for successful repurposing against emerging viral infections. Since the viral envelope is derived from host cell membranes rather than encoded by the viral genome, there is no obvious way for resistance development to membrane-targeting drugs.

This review has examined various mechanisms of membrane-targeted antiviral activity, each presenting distinct advantages and limitations. Photosensitizers demonstrate exceptionally broad-spectrum antiviral potency, yet frequently suffer from poor solubility and bioavailability. Furthermore, their light-dependent activation complicates treatment of systemic infections. While developing near-infrared-absorbing photosensitizers or nanoparticle formulations partially mitigates these solubility and activation constraints, challenges remain. Similarly, molecular tweezers exhibit high antiviral activity comparable to photosensitizers. They function in both light and dark conditions, though their aromatic frameworks inherently limit aqueous solubility and consequent bioavailability.

Protein-based compounds—including antimicrobial peptides, labyrinthopeptides, and peptidomimetics—often display favorable bioavailability and low cytotoxicity. However, their antiviral efficacy is frequently modest (EC_50_ ~50 μM), coupled with complex and costly production processes that impose significant practical limitations.

Host cell-targeting agents inducing ER stress (e.g., thapsigargin) are promising candidates for broad-spectrum antiviral development. Thapsigargin combines negligible cytotoxicity with high antiviral activity and natural sourcing potential despite structural complexity. Nevertheless, its poorly characterized mechanism of action and metabolic pathways preclude definitive conclusions about clinical applicability.

Currently, most membrane-targeting agents remain in preclinical (in vitro/in vivo) testing stages. The inherent limitations of each mechanism currently prevent progression to clinical trials. Analysis of existing data on membrane-active agents suggests potential benefits from combining multiple membrane-targeting compounds or integrating other antiviral mechanisms within a single molecule. This approach could enhance the drug’s spectrum of activity and significantly delay the emergence of viral resistance. This approach would enable synergistic combination of compounds with distinct antiviral mechanisms, simultaneously leveraging their therapeutic benefits while circumventing individual limitations.

## Figures and Tables

**Table 1:** Examples of antiviral compounds that inhibit different stages of the viral life cycle.

Inhibition Stage	Compound	Class	Mechanism	Research Stage
Attachment	Palivizumab	Antibody	Blocking the binding of the virus to cell receptors	Approved and in use
	AEEA-16	Peptidomimetic		Preclinical trials
Fusion	Maraviroc	Small molecule combining various functions	Blocking fusion proteins	Approved and in use
	LJ-oo1	Photosensitizer	Membrane damage	Preclinical trials
	CLR01	Molecular tweezers	Membrane destruction	
Entrance	Amantadine	Cage hydrocarbon amine	Ion channel blocker	Approved and in use
Transcription/replication	Remdesivir	Nucleoside	RNA polymerase inhibitors	Approved and in use
	Favipiravir	Nucleoside analogue		
	Lopinavir	Peptidomimetic	Protease inhibitors	
	Nirmatrelvir	Peptidomimetic		
Reverse transcriptase inhibitors	Lamivudine	Nucleoside	DNA chain break	Approved and in use
	Efavirenz	Benzoxazinone	Allosteric inhibition of enzymes	
Virion assembly	Thapsigargin	Terpene	Activation of EPR stress	Preclinical trials
	Tunicamycin	Nucleoside		
Virion release	Oseltamivir	Small molecule combining various functions	Neuraminidase inhibition	Approved and in use
	Baloxavir	Small molecule combining various functions	Hemagglutinin inhibition	
Immunomodulators	Interferon-α	Protein	Activation of immunity	Approved and in use

**Table 2:** Examples of enveloped, quasi-enveloped, and non-enveloped viruses.

Virus	Type	Genetic Material
Tick-borne encephalitis virus (TBEV)	Enveloped	(+)ssRNA
Herpes simplex virus (HSV)	Enveloped	dsDNA
Vesicular stomatitis virus (VSV)	Enveloped	(−)ssRNA
Influenza virus A (IVA)	Enveloped	(+)ssRNA
Zika virus (ZIKV)	Enveloped	(+)ssRNA
Human immunodeficiency virus (HIV)	Enveloped	(+)ssRNA
Rift Valley fever virus (RVFV)	Enveloped	(−)ssRNA
Hepatitis A virus (HAV)	Quasi-enveloped	(+)ssRNA
Hepatitis E virus (HEV)	Quasi-enveloped	(+)ssRNA
Human papilloma virus (HPV)	Non-enveloped	dsDNA
Rotavirus A (RVA)	Non-enveloped	dsRNA
Human rhinovirus A (HRV-A)	Non-enveloped	(+)ssRNA
